# Procrastination as a Threat to the Psychological Security of the Educational Environment

**DOI:** 10.3390/bs10010001

**Published:** 2019-12-18

**Authors:** Anna Litvinova, Alexey Kokurin, Valentina Ekimova, Anna Koteneva, Vyacheslav Pozdnyakov

**Affiliations:** Department of Scientific Bases of Extremal Psychology Moscow State University of Psychology and Education, Sretenka ul., 29, 127051 Moscow, Russia; kokurin1@bk.ru (A.K.); iropse@mail.ru (V.E.); akoteneva@yandex.ru (A.K.); pozdnyakov53@mail.ru (V.P.)

**Keywords:** procrastination, academic motivation, motivation of academic, intellectual and professional activity, interpersonal relations, psychological security of the educational environment

## Abstract

The present study provides an analysis of the concept of procrastination and its features, together with a discussion of the fundamental approaches to its investigation. It examines procrastination as a threat to the psychological security of the educational environment. The author presents the results of an empirical investigation of the characteristics of interpersonal relations in the educational environment, academic motivation, and general motivation of students with various degrees of procrastination. The subjects of the investigation were 95 students, of the average age of 18.2 years, in an institution of higher education dedicated to the humanities. The subjects were evaluated using the procrastination scale for student populations developed by C. Lay, the academic motivation scale of R.J. Vallerand, the self-evaluation survey of motivation of academic, intellectual, and professional activity developed by N.A. Bakshaeva and A.A. Verbitsky, and the “Survey of Interpersonal Relations in an Educational Environment” developed by G.S. Kozhukhar and V.V. Kovrov. The primary hypothesis of the study, that the interconnections of interpersonal relations in an educational environment with academic and general motivation would differ in the groups of students with different levels of procrastination, was confirmed. The differences in the evaluations of the quality of interpersonal relations, and indicators of academic and intellectual motivation of students with different levels of procrastination were of special particular significance. It was shown that, the higher the level of students’ procrastination, the greater the interaction between the negative aspects of interpersonal relations in the educational environment and the external academic motivation.

## 1. Introduction

In the current environment of rapid transformation in a digital society and the multitude of demands on the individual, interest in the study of procrastination as a phenomenon is rising. Procrastination is understood as conscious delay, failure to complete, or postponement of planned activities, accompanied by a sense of internal discomfort and a negative emotional state, connected with an expectation of negative consequences, according to P. Stil, C. Lay, A. Ellias, et al. [1]. In Russian psychology, the terms otkladyvanie (“postponement”) or promedlenie (“delay”) are used as synonyms for “procrastination.” Procrastination may appear in any sphere of human activity: Academic, work, social (postponement of meetings or phone calls), professional, or home. According to V. S. Kovylina, 46 to 95 percent of students regard themselves as procrastinators [2]. Procrastinators often delay realizations of their plans until “later” and start to work on them only at a time when there is no point in attempting to make up for lost time. This is precisely the way in which the negative consequences of such postponement manifest themselves. In addition to external unpleasant results, procrastination is characterized by significant emotional discomfort: A sense of guilt, worry, and uncertainty as to whether the situation will be resolved positively, all of which rise as a deadline approaches [3]. Worry increases, not only because of the temporary and high expectations of others, but because of the increased self-imposed demands for results by the procrastinator. Emotional discomfort, negative subjective feelings, and a general dissatisfaction with one’s own activity are fundamental psychological signs of procrastination.

One of the problems of this phenomenon is the vagueness of the boundary between “procrastination” and “laziness”. E.P. Ilin distinguishes two types of procrastinators: “tense” ones are more “nervous”, “upset”, “subject to a sense of guilt”, “relaxed procrastinators” categorizable in nontechnical language as “lazy”, are concerned only with completing those tasks that will bring pleasure at the present moment, “here and now”. In this case, laziness is a form of procrastination [4]. Yet in spite of similar behavior, associated with the failure to complete tasks, a procrastinator is distinguished from a lazy person by a negative emotional experience. Rather than being indifferent, procrastinators are actively interested in achieving an expected result. The fundamental differences between the two types lies in the subjective emotional suffering of the individual with reference to the delay of planned tasks.

V.S. Kovylin distinguishes the two types of procrastination as expressions of the emotional reactions regarding planned or necessary activities. In the case of “relaxed procrastination,” the subject spends time on other, more pleasant activities and recreations. “Tense” procrastination is connected with a loss of a sense of time, a dissatisfaction with oneself, vague life goals, indecision and feelings of insecurity [2]. The basic types of procrastination have been characterized by N. Milgram, J. Batory, and D. Mourer: Daily, or Household: Daily or everyday (postponement of tasks which should be done every day), procrastination in decision making, including minor decisions; neurotic procrastination (postponement of important decisions); compulsive, which includes both everyday procrastination and postponement of decisions; and academic, postponement of assignments and preparation for examinations [2]. A habit of postponing things until later may lead to lower academic standing, heightened nervousness, the formation of an inferiority complex, and problems in interactions with others. The majority of students do not deny that they postpone the completion of academic assignments.

Researchers have noted that procrastination is most widespread among students. Procrastination among students has been studied in connection with anxiety [5,6,7], self-esteem [8,9], defense mechanisms and coping strategies [10,11,12], perfectionism [13,14,15] etc. Here, we are concerned here with the specific interactions of procrastination with motivation.

R. Klassen et al. have shown that academic procrastination, as a tendency on the part of students to postpone until later, is linked to a low effectiveness of self-motivation. K. Sinekal, with a group of researchers, have concluded that intrinsic motivation associated with the content of activities and identified motivation (motivation to perform personally significant tasks) are typical for students with a lower level of procrastination, while extrinsic motivation and demotivation are typical for students with a higher level of procrastination [2].

T.V. Zaripova and N.A. Danilova have examined the interconnections between students’ academic procrastination and academic motivation. Their results have demonstrated that students whose academic motivational structures are dominated by internal motivators (intellectual curiosity, professional drive) and positive motivators (desire for success) are less prone to academic procrastination. Academic procrastination is not connected with the level of social motivators for the academic activity of the students [16]. Students with an expressed manifestation of procrastination are characterized by a predominance of negative motivation for academic activity (avoidance of failure) over positive (achievement of success). T. M. Tron asserts that “active procrastinators” are distinguished by weak academic motivation and low academic success [17].

Researchers have suggested that the process of studying is characterized by conditions under which procrastination often arises. O.O. Shemyakina points to the following conditions of the academic process that provoke procrastination: The external origin of assignments; delayed consequences; long periods given for completion; boring, routine, unpleasant assignments; pressure from other obligations; delayed gratification and rewards; any written assignments [18]. Procrastination may allow students to avoid direct evaluations of their performance, but the resulting obligation to complete them in a short time may prevent them from realizing their potential. Such situations lead to negative consequences, but it may support self-esteem at the necessary level [19].

Procrastination that arises in the context of learning may lead to damage to the psychological security of the educational environment. By the psychological security of the educational environment, we mean conditions that allow the high level of satisfaction, sense of security, comfort, and socio-psychological competence that are necessary for the realization of individual potential in a variety of areas [20]. I.A. Baeva suggests that the interpersonal relations of inhabitants of the learning environment “give birth to” the psychological security of that environment [21]. A satisfactory level of personally trusting social interaction is one of the criteria for psychological security of the educational environment of contemporary educational institutions [21,22]. Constant delaying and postponing of required assignments by students may lower the level of satisfaction with interpersonal relationships with inhabitants of the educational environment.

In modern psychology, a deficit of scientific knowledge about procrastination as a risk of the psychological safety of the educational environment has been identified. To fill this deficit, an empirical study has been conducted, which aimed at identifying the connection of interpersonal relationships with motivation and motives of students with different levels of procrastination.

We have hypothesized that the interconnections of interpersonal relationships in the educational environment with academic and general motivation are different for groups of students with different levels of procrastination. In order to test this hypothesis, we set forth the following sub-hypotheses: (1) There are differences in the evaluations of the quality of interpersonal relationships between students with different levels of procrastination, which can determine the psychological security of the educational environment. (2) There are differences between the indicators for academic and general intellectual motivation for students with different levels of procrastination. (3) The higher the level of procrastination, the greater the linkage of negative qualities of interpersonal relationships in the educational environment with external academic motivation.

## 2. Methods

### 2.1. Participants and Procedure

The research procedure consisted of students filling out the questionnaire individually. The questionnaires were sent to full-time students in three faculties of the Moscow State Psychological and Pedagogical University. Answers were received with a coefficient of 58% (23% of the questionnaires were incomplete, with 15% of students no contact was established, and 4% refused to participate in the study). This percentage is considered sufficient, since the study was conducted on an anonymous and voluntary basis. As a result, 95 questionnaires were filled out (72% were women, average age 19.3 years). Data was collected and processed in 2018.

### 2.2. Methods

The method of comparative analysis was used. The subjects were evaluated on the scale of procrastination developed by K. Ley [23], validated for Russian samples [24], the scale of academic motivation developed by R.J. Valleran [25], a self-assessment survey of the motivation of educational, intellectual and professional activity, developed by N.A. Bakshaeva and A.A. Verbitsky [26], as well as the questionnaire, “The quality of interpersonal relations in the educational environment,” developed by G.S. Kozhuharom and V.V. Kovrov [27].

The reliability and validity of the research results is ensured by a sufficient volume of the examined sample; the use of a set of methodological procedures adequate to the object, subject, goal and objectives of the study, using methods of quantitative analysis.

The scale of procrastination developed by K. Ley [23] allows you to determine the level of procrastination by quantitative indicators from 20 to 100: 20–45 is low level, 46–60 is average level, and 61–100 is a high level of procrastination. The Academic Motivation Scale by R.J. Vallerana [25] in the modification of Gordeeva, Sychev and Osin, allows reliable assessing of qualitatively different types of internal (cognitive motivation, achievement and self-development), external motivation (self-esteem motivation, introjected, external) and motivation in accordance with the obtained points. Self-assessment survey of the motives of educational, intellectual and professional activity, according to N.A. Bakshaeva and A.A. Verbitsky [26], evaluates current motives: Educational, cognitive, and professional; the adoption of which is evaluated by quantitative indicators from 8 to 40, with the higher the score, the higher the adoption of the corresponding motivation. The questionnaire, “The quality of interpersonal relations in the educational environment,” by G.S. Kozhukhar and V.V. Kovrova [27] includes 8 scales: The scale of trust, aggressiveness, goodwill, conflict, acceptance, hostility, tolerance, and manipulative attitude. The judgments of the questionnaire are evaluated from 0 to 4 points, in accordance with the key points, and are calculated on each scale, which reveal the features of positive and negative attitudes in the system of interpersonal interaction. To analyze the obtained data, we used frequency analysis, descriptive statistics, non-parametric Mann–Whitney U test to identify significant differences, and the Kendall Tau-b correlation coefficient to determine significant correlations. To calculate the data, the IBM SPSS Statistics v.20 program was used.

The analysis of the data obtained in the study was carried out in 4 stages:

1. The selection of groups with different levels of procrastination.
2. Determination of the severity and significant differences of indicators by methods in groups with different levels of procrastination.
3. Determination of significant correlations of the qualities of interpersonal relations in the educational environment with academic motivation and motives of educational, intellectual and professional activity in groups with different levels of procrastination.
4. Analysis of the results.

## 3. Results

### 3.1. Determination of the Level of Student Procrastination

Students were divided into two groups with different levels of procrastination: Moderate and high, on the basis of the C. Lay scale of procrastination.

Table 1 shows that 46.77% of the students exhibited a moderate level of procrastination (group 1), while 53.3% of the students exhibited a high level of procrastination. There were no students with a low level of procrastination.

On the basis of the Mann–Whitney criteria, a significant difference in the indicators of procrastination was found (Table 2).

Indicators of procrastination were significantly apparent in the second group of students, who were distinguished by a weak focus on completion of high-priority tasks and postponement of such tasks to an undetermined time.

### 3.2. Characteristics of Interpersonal Relations in the Educational Environment and Motivation of Students with Various Levels of Procrastination

Further, an analysis was made of the results of the descriptive statistics and estimations of significant differences in interpersonal relationships in the educational environment, and of the motivation of students with varying degrees of procrastination. Descriptive statistics of the characteristics of the two groups in the quality of interpersonal relations in the educational environment are presented in Table 3.

The results indicate that students with a moderate level of procrastination feel that interpersonal relationships in the educational environment are characterized by acceptance, goodwill, aggression, and manipulative relationships. Students with a high level of procrastination believe that interpersonal relationships in the educational environment are characterized by goodwill, acceptance, aggression, proneness to conflict, and manipulative relationships.

It has been shown that according to the Mann–Whitney criterion that significant differences between groups are observed only for the indicator of proneness to conflict (*p* < 0.05) for interpersonal relationships (Table 4).

Indicators of academic and general motivation for students of the two groups are presented in Table 5.

Students with a moderate level of procrastination show a greater expression of intellectual motivation, motivation for personal growth and self-esteem, as well as professional and academic motivation. For students with a high level of procrastination, the order is somewhat different. For them, the sequence of intellectual motivation, motivation for personal growth, introjected motivation, professional, intellectual, and academic motivation is characteristic.

Significant differences on the scale of academic motivation among students of different groups are contained in Table 6. It has been shown that students with a moderate degree of procrastination are significantly distinguished by the expression of professional (*p* < 0.05), academic (*p* < 0.05) and cognitive (*p* < 0.05) activity. In students with a high degree of procrastination, introjected motivation (*p* < 0.01) and amotivation (*p* < 0.05) predominate.

### 3.3. Characteristics of Interconnections between Interpersonal Relations in the Educational Environment and Academic and General Motivation among Students with Different Levels of Procrastination

The coefficients of correlation have been calculated for the analysis of the interconnections between the attributes of interpersonal relations in the educational environment and academic and general motivation. The results testify to the presence of significant interconnections between the attributes of interpersonal relations in the educational environment and academic motivation (Table 7). Significant interconnections between the attributes of interpersonal relations in the educational environment with cognitive and professional motivation were not found.

In the group of students with a moderate level of procrastination, correlations were found with relationships of goodwill in the educational environment: Positive, with the motivation of personal growth (*p* < 0.05), negative, with introjected (*p* > 0.05) and external (*p* < 0.05) motivation. A negative correlation was found between acceptance in interpersonal relations and amotivation (*p* < 0.05). Correlations were found for tolerance of relations in the educational environment: Negative, with cognitive motivation (*p* < 0.05) and positive, with introjected motivation (*p* < 0.05). Manipulative relationships in the educational environment were significantly negatively correlated with the cognitive motivation of students (*p* < 0.01).

In the group of students with a high level of procrastination, a negative correlation was found between goodwill in relationships in the educational environment and cognitive motivation (*p* < 0.05). Correlations were found between acceptance in interpersonal relationships: A negative one with motivation to accomplish (*p* < 0.05), and a positive one with motivation for self-esteem (*p* < 0.05). Positive correlations were found between tolerance of relations in the educational environment and introjected (*p* < 0.05) as well as external motivation (*p* < 0.05). Positive correlations were found between hostile relations in the educational environment and introjected (*p* < 0.05), external motivation (*p* < 0.01), and amotivation (*p* < 0.01). Positive correlations were found between manipulative relationships in the educational environment with external motivation (*p* < 0.01) and amotivation (*p* < 0.01). Positive relationships in the educational environment were negatively connected with motivation to accomplish (*p* < 0.01) and positive relationships were positively correlated with amotivation (*p* < 0.01).

## 4. Discussion and Conclusions

According to the results obtained, students with a low level of procrastination were not found. 46.7% of students were characterized by moderate procrastination, and 53.3% were characterized by a high level of procrastination, thus demonstrating the tendency of students to delay in decision-making and postponement of completion of various tasks until later.

Analysis of the quality of interpersonal relations in the educational environment of the institution showed that the majority of students surveyed take a positive view of the relationships that they have, and sense a goodwill in interpersonal relationships in the educational environment, acceptance, and a potential for personal growth. On the basis of this study, it is possible to assert the existence of a psychologically secure educational environment on the level of the database. Nevertheless, it became apparent that students sense the existence of negative aspects of interpersonal relationships in the educational environment, which may pose risks of breaches to its security. Students with different levels of procrastination feel that interpersonal relationships in the educational environment are characterized by aggressivity and manipulation. It is characteristic that, based on the results of nonparametric comparison of students with a high level of procrastination evaluations, significant expressions of proneness to conflict in interpersonal relations in the educational environment is dedicated.

Differences were found in the indicators of academic motivation and motivation of academic and cognitive activity among students with various levels of procrastination. Students with moderate procrastination are significantly distinguished as motivated by professional, academic, and cognitive activity, which causes them to strive to acquire professional knowledge, skills, and abilities appropriate to their profession. External introjected motivation, which manifests itself in feelings of obligation and shame in relation to oneself and significant others, as well as amotivation in the form of lack of interest and sense of meaning in academic activity, prevail in students with a high level of procrastination. These results correspond with the conclusions about the interconnections of academic procrastination and student motivation; for students with expressed symptoms of procrastination, negative motivation for academic activity (avoidance of failure) is more common than positive motivation (striving for success) [16,24].

The coherence of the results, obtained with the majority of those of students’ procrastination investigations [2,8,9,16,17,18,20], allowed us to state that they could be not restricted to the limited sample of a concrete university but distributed to the entire population of higher education students, and to continue the investigation differentiating between the students with different levels of procrastination.

The results of the correlation analysis prove that the more students with moderate procrastination value goodwill in relationships in the educational environment, the higher their internal motivation for personal growth is directed at the development of abilities, academic potential, and achievement of professional competence, and the lower their introjected and external motivation. It may be also concluded that the more they value the acceptance of interpersonal relations, the lower their amotivation. It is important to note that expressions of goodwill and acceptance of interpersonal relations in the educational environment raise the psychological security of the educational environment and inspire students of this group to personal development. On the other hand, the greater the value placed on tolerance in interpersonal relations, the lower students’ cognitive motivation and the higher their introjected motivation; and the more often manipulative relationships manifest themselves in the educational environment, the lower the cognitive motivation of the students. In this group, tolerance and manipulative relationships lead to a lowering of the psychological security of the educational environment, accompanied by a lowering of cognitive motivation and a raising of external motivation.

It has been shown, that the more students with high procrastination value goodwill in interpersonal relations in the educational environment, the less they express cognitive motivation, which drives students to learn new things, to understand the subjects that they are studying, and to find pleasure in the process of learning. The more they value the acceptance of interpersonal relationships, the lower their internal motivation to achieve the best results in their studies, and the higher their external motivation for self-esteem, and their desire to study for the sake of their own significance. Such negative characteristics of interpersonal relationships as hostility and manipulation are positively connected with introjected and external motivation, and amotivation.

Therefore, for the first time, the evidence of interconnection between procrastination and psychological security of the educational environment was obtained, and a different connection between interpersonal relationships in the educational environment with academic motivation and motives of activity in groups of students with different levels of procrastination were evaluated and analyzed. It has been found that students with a high level of procrastination often show their negative qualities in interpersonal relationships, provoking violation of the psychological safety of the educational environment. Besides that, the more the procrastinators valued their interpersonal relations, the lower their cognitive and internal motivation was.

Further research of procrastination as a threat to the psychological security of the educational environment, with particular attention to the characteristics of interpersonal relations and motivation of students, is undoubtedly required. In order to safeguard the psychological security of the educational environment in the institutions of higher education, it should be recommended to develop and to realize the targeted psychological intervention programs aimed at lowering the level of students’ procrastination.

## Figures and Tables

**Table 1:** Indicators of students’ procrastination.

	1st Group: Moderate Procrastination		2nd Group: High Procrastination	
	M	Sd	M	Sd
Procrastination	52.62	4.28	67.67	5.37

**Table 2:** Empirical value of Mann–Whitney U criterion when comparing indicators.

	1st Group: Moderate Procrastination		2nd Group: High Procrastination	
Procrastination	-		231 **	

Note: ** *p* < 0.01.

**Table 3:** Indicators of the quality of interpersonal relationships in the educational environment among students with different levels of procrastination.

	1st Group: Moderate Procrastination		2nd Group: High Procrastination	
	M	Sd	M	Sd
Trust	5.95	1.69	6.46	1.14
Goodwill	7.48	1.50	8.17	1.83
Acceptance	7.62	1.75	8.13	1.48
Tolerance	3.52	1.99	3.38	1.71
Aggression	6.38	2.29	6.88	1.87
Proneness to conflict	5.62	1.69	6.79	1.28
Hostility	5.43	2.69	5.38	3.23
Manipulative relationships	6.24	1.55	6.54	1.64
Positive relationships	6.14	0.65	6.53	0.84
Negative relationships	5.92	1.37	6.40	1.20

**Table 4:** Empirical value of Mann–Whitney U, “Survey of Interpersonal Relations in an Educational Environment,” developed in student groups with different levels of procrastination.

	1st Group: Moderate Procrastination	2nd Group: High Procrastination
“Survey of Interpersonal Relations in an Educational Environment” developed		
Proneness to conflict	-	146 *

Note: * *p* < 0.05.

**Table 5:** Indicators of academic and general motivation among students with various levels of procrastination.

	1st Group: Moderate Procrastination		2nd Group: High Procrastination	
	M	Sd	M	Sd
*The academic motivation scale*				
Intellectual motivation	16.48	1.69	15.58	3.12
Motivation to accomplish	13.19	3.37	12.38	2.45
Motivation for personal growth	15.38	2.69	15.17	2.43
Motivation for self esteem	13.29	2.78	13.54	3.30
Introjected motivation	12.57	2.79	14.71	2.26
External motivation	11.33	3.29	13.04	2.97
Amotivation	6.71	2.35	8.83	3.02
*The motives of educational, cognitive and professional activities*				
Academic motivation	33.10	3.83	29.00	6.03
Cognitive motivation	32.14	4.76	29.38	5.07
Professional motivation	34.19	5.37	31.00	5.53

**Table 6:** Empirical significance of the Mann–Whitney criteria comparing indicators in student groups with different levels of procrastination.

	1st Group: Moderate Procrastination	2nd Group: High Procrastination
*The academic motivation scale*		
Introjected motivation	-	138 **
Amotivation	-	149.5 *
*The self-evaluation survey of motivators of academic, cognitive, and professional activity developed*		
Academic motivation	149 *	-
Cognitive motivation	158 *	-
Professional motivation	152 *	-

Note: ** *p* < 0.01; * *p* < 0.05.

**Table 7:** Results of a correlative analysis by Kendall’s tau coefficient.

The Survey of Interpersonal Relations in an Educational Environment	The Academic Motivation Scale	1st Group: Moderate Procrastination	2nd Group: High Procrastination
Goodwill	Motivation for personal growth	0.398 *	
	Introjected motivation	−0.379 *	
	External motivation	−0.393 *	
	Cognitive motivation		−0.332 *
Acceptance	Amotivation	−0.472 **	
	Motivation to accomplish		−0.335 *
	Motivation for self esteem		0.348 *
Tolerance	Cognitive motivation	−0.354 *	
	Introjected motivation	0.440 *	0.403 *
	External motivation		0.405 *
Hostility	Introjected motivation		0.352 *
	External motivation		0.439 **
	Amotivation		0.423 **
Manipulative relationships	Cognitive motivation	−0.444 **	
	External motivation		0.488 **
	Amotivation		0.431 **
Positive relationships	Motivation to accomplish		−0.424 **
Negative relationships	Amotivation		0.428 **

Note: * the correlation is significant at the level *p* < 0.05; ** the correlation is significant at the level *p* < 0.01.
